# Global Metabolic Profiling of Plasma Shows that Three-Year Mild-Caloric Restriction Lessens an Age-Related Increase in Sphingomyelin and Reduces L-leucine and L-phenylalanine in Overweight and Obese Subjects

**DOI:** 10.14336/AD.2016.0330

**Published:** 2016-12-01

**Authors:** Minjoo Kim, Sang-Hyun Lee, Jong Ho Lee

**Affiliations:** ^1^National Leading Research Laboratory of Clinical Nutrigenetics/Nutrigenomics, Department of Food and Nutrition, College of Human Ecology, Yonsei University, Seoul, 03722, Korea; ^2^Department of Food and Nutrition, Brain Korea 21 PLUS Project, College of Human Ecology, Yonsei University, Seoul, 03722, Korea; ^3^Department of Family Practice, National Health Insurance Corporation Ilsan Hospital, Goyang, 10444, Korea; ^4^Research Institute of Science for Aging, Yonsei University, Seoul, 03722, Korea

**Keywords:** mild-calorie diet, BMI, sphingomyelin, L-leucine, L-phenylalanine

## Abstract

The effect of weight loss from long-term, mild-calorie diets (MCD) on plasma metabolites is unknown. This study was to examine whether MCD-induced weight reduction caused changes in the extended plasma metabolites. Overweight and obese subjects aged 40-59 years consumed a MCD (approximately 100 kcal/day deficit, *n*=47) or a weight-maintenance diet (control, *n*=47) in a randomized, controlled design with a three-year clinical intervention period and plasma samples were analyzed by using UPLC-LTQ-Orbitrap mass spectrometry. The three-year MCD intervention resulted in weight loss (-8.87%) and significant decreases in HOMA-IR and TG. The three-year follow-up of the MCD group showed reductions in the following 13 metabolites: L-leucine; L-phenylalanine; 9 lysoPCs; PC (18:0/20:4); and SM (d18:0/16:1). The three-year MCD group follow-up identified increases in palmitic amide, oleamide, and PC (18:2/18:2). Considering the age-related alterations in the identified metabolites, the MCD group showed a greater decrease in L-leucine, L-phenylalanine, and SM (d18:0/16:1) compared with those of the control group. Overall, the change (Δ) in BMI positively correlated with the ΔTG, ΔHOMA-IR, ΔL-leucine, and ΔSM (d18:0/16:1). The ΔHOMA-IR positively correlated with ΔTG, ΔL-leucine, ΔL-phenylalanine, and ΔSM (d18:0/16:1). The weight loss resulting from three-year mild-caloric restriction lessens the age-related increase in SM and reduces L-leucine and L-phenylalanine in overweight and obese subjects. These changes were coupled with improved insulin resistance (ClinicalTrials.gov: NCT02081898).

The World Health Organization and the International Obesity Task Force recommended lower BMI cutpoints for defining overweight and obesity in Asian populations [1]. Some prospective studies have examined the association between body weight and mortality in Asian populations; Yuan et al. [2] found a U-shaped relationship between BMI and mortality which shows high relative risk with a BMI of less than 18.5 and a BMI 26.0. In a similar fashion, Zhou [3] reported an age-adjusted mortality was higher in subjects with a BMI of less than 18.5 or 28.0 or more. Using participants with a BMI of 24.0-24.9 as the reference group, the relative risks of mortality increase in a BMI of lower and upper than reference criteria [4] which is consistent with previous studies. These findings support the use of a single common recommendation for defining overweight and obesity in all racial and ethnic groups.

There has been growing evidence that obesity-related conditions are characterized by a broad perturbation of the metabolic physiology involving considerable changes in the metabolism of amino acids, fatty acids [5-8], and glucose [9]. This new evidence motivates the application of methods such as metabolomics, which is designed to monitor a broad range of molecular species, to study the beneficial effects of potentially health-promoting diets [8, 10]. Branched-chain amino acids (BCAAs) are noteworthy in the context of experimental and clinical data. It has been suggested that these amino acids may be both the markers and effectors of insulin resistance (IR) [6, 11, 12] and can predict future diabetes [13, 14]. BCAAs have recently been linked to the risks of developing hyperglycemia and diabetes in older populations [13, 15, 16]; Würtz et al. [14] suggested that these amino acids mediate the risk for future diabetes because they are markers for the development of IR. However, the ways in which these amino acids mediate the risk of developing IR and diabetes remain incompletely understood. Recently, Gu et al. [5] assessed the effects of diet in obese subjects and found that the BCAA level did not change significantly after 4 or 8 weeks of very low-calorie diets (VLCD; < 800 kcal intake/day). This finding indicates that plasma BCAA concentrations are likely unaffected by VLCD. By contrast, an energy-restricted diet (-15% of daily energy requirements) in overweight and obese older adults over an 8-week period produced significant weight reduction (7%), an improvement in glucose and lipid profiles, and a decrease in isoleucine [17].

The effect of weight loss when mediated by a long-term mild-calorie diet (MCD) on plasma metabolites is unknown. Knowledge of the biochemical effects of long-term MCD-induced weight loss on circulating metabolites may be valuable for identifying specific metabolites that directly or indirectly affect insulin sensitivity. We expected to investigate what metabolic changes occurred during weight loss when it was induced by long-term MCD in overweight and obese subjects, and we considered that the observed changes could be possible biomarkers for predicting the future risk of a disease arising under overweight and obese conditions, facilitating the design of early interventions to prevent disease progression. In our previous study, we suggested that changes in certain metabolites precede IR during periods of elevated alanine aminotransferase [18], providing novel insights into the metabolic alterations that occur during the early metabolic stages of disease. Likewise, we hypothesize that long-term MCD-induced weight loss affects the plasma metabolic profile, and that the observed profile changes may provide new clues about the beneficial effects of weight loss induced by following a 100 kcal/day deficit for three years. Therefore, the objective of this study was to examine whether the reduction induced by MCD (an approximately 100 kcal/day deficit) changed the extended plasma metabolite profile in middle aged overweight and obese subjects during a three-year intervention study.

## MATERIALS AND METHODS

### Study populations

120 overweight and obese subjects [25 ≤ body mass index (BMI) ≤ 34 kg/m^2^] aged 40-59 years were recruited at a health-promotion center in Ilsan Hospital, Korea, from June to August 2010. Exclusion criteria were subjects who had cardiovascular disease, renal disease, thyroid disease, inflammatory disease, cancer; women who were pregnant; taking blood pressure (BP), lipid, glucose-lowering medications or supplements at the baseline and during the three-year follow-up. The subjects who participated in weight-reduction programs within the last three years were also excluded. The written informed consent was obtained before study participation and the protocol was approved by the institutional review board of Yonsei University and Ilsan Hospital according to the Helsinki Declaration.

### Study protocol and energy intake management

The participated subjects were allocated into two groups according to independently performed computer randomization. The duration of the study was three years and the program goal for the MCD group was to achieve an approximately 5% of weight loss from their initial body weight. Over the study period, each participant’s MCD was mildly caloric-restricted with an approximately 100 kcal/day deficit. Participants were recommended to take out 1/3 of a bowl of rice from one meal a day for an easier application of 100 kcal deficits, given that the calories in a bowl of rice is 300 kcal, according to the food-composition tables from the Rural Development Administration (8^th^ Ed., 2011) of Korea. The usual dietary intake was recommended to the control group. The trained dietitian supported dietetic education to participants every month for the first 6 months, every 3 months for the next 15 months, and every 5 months for the subsequent 15 months. Participants were offered phone support with a dietitian and they received general information regarding diet and lifestyle self-management during the study period.

All participants were asked to maintain their ordinary dietary intake for seven days before their visits and were required to submit a food journal at the baseline visit (before group allocation) and after group allocation, they submit a food journal at every 3 months. The food journal covered 3-day dietary record which is consisted of 2 weekdays and 1 weekend day. The participants were told to drink no more than one alcoholic beverage (15 g alcohol) per day. Amount of food was measured by using standard measuring cups, spoons, and weights in grams; the input accuracy of the food journal was confirmed by semi-quantitative food-frequency questionnaires. Nutrient intake was determined and calculated as a mean value from a 3-day dietary record by using the Computer-Aided Nutritional Analysis Program (CAN-pro 3.0, Korean Nutrition Society, Seoul, Korea). Good compliance with dietary interventions was defined as a reduction in the mean food intake value for 3 days of at least 100 kcal at each time point from the baseline. Physical activity was assessed from activity patterns with a mean value from a 3-day record (2 weekdays and 1 weekend day) [19] and total energy expenditure (TEE) was calculated by the Harris-Benedict equation [20].

### Anthropometry and blood collection

To calculate BMI (kg/m^2^), body weight and height were measured in the morning with participants unclothed and without shoes. Waist circumference was measured at the umbilical level at the end of normal expiration while standing. BP was measured in both arms by an automatic BP monitor (TM-2654, A&D, Tokyo, Japan) after a 20-min rest. Venous blood specimens were collected in EDTA-treated and plain tubes after a 12-h fast. Serum and plasma were withdrawn after centrifugation for 30 min at 1,230 g and 4?, and aliquots were then stored at -70? until analysis.

### Biochemical parameters

The lipid profile including total-cholesterol, triglyceride (TG), and free fatty acid (FFA) were measured using a Hitachi 7600 autoanalyzer (Hitachi Ltd., Tokyo, Japan). For separating HDL-cholesterol by dextran-sulfate magnesium precipitation the enzymatic method was used. The LDL-cholesterol concentration was calculated by the Friedewald equation. Lipoprotein-associated phospholipase A_2_ (Lp-PLA_2_) activity was measured with a high-throughput radiometric activity assay [21]. The fasting glucose level was analyzed by hexokinase method with a Hitachi 7600 autoanalyzer. Insulin level was measured with immunoradiometric assay, using commercial kit provided by Immuno Nucleo Corporation (Stillwater, MN, USA) and IR was calculated by homeostasis-model assessment (HOMA).

### Global (non-targeted) metabolic profiling

The method for sample preparation, analysis, using an ultra-performance liquid chromatography (UPLC) and linear-trap quadrupole (LTQ) Orbitrap XL mass spectrometry (MS), data processing, and identification of plasma metabolites were demonstrated in previous study [19].

### Statistical analysis

Statistical analyses were performed with SPSS v. 21.0 (IBM/SPSS, Chicago, IL, USA) and a two-sided *P*-value of < 0.05 was considered statistically significant. Differences in variables between the two groups at the baseline and the three-year follow-up were tested with a Student’s independent *t*-test. Applying the general linear model is accomplished by adjust for baseline values to compare changes in variables between the two groups. Paired *t*-test was used for evaluating differences between two time-points in each group. *Pearson’s* and *partial* correlation coefficients were used to examine the relations between variables over time. Multiple regression analysis was performed to identify major plasma metabolites that were correlated with weight changes. False discovery rate adjusted *q*-values were computed using the fdrtool package in R version 3.1.2. Heat map was created to visualize and evaluate relations among metabolites and other variables in study populations. SIMCA-P+ software version 12.0 (Umetrics, Umeå, Sweden) was used for performing a multivariate analysis on plasma metabolites [19]. To classify the discrimination between the two groups, a partial least-squares discriminant analysis (PLS-DA) was applied for visualizing the score plot with the first- and second-PLS components.

## RESULTS

At the endpoint of dietary intervention, 26 participants were removed from the study. This group included 7 who declined further participation, 11 who showed poor compliance, 5 who were diagnosed with a disease, and 3 who were removed from the study for personal reasons.

### Clinical characteristics and energy intake

There were no significant differences between the control group and the MCD group in the following baseline characteristics: gender (25 males and 22 females in both groups), age (control, 48.28±1.05 years; MCD, 49.64±1.13 years), body weight (control, 74.46±1.51 kg; MCD, 76.35±1.50 kg), BMI (control, 27.54±0.22 kg/m^2^, range: 25.50 ~ 31.27 kg/m^2^; MCD, 27.78±0.28 kg/m^2^, and range: 25.00 ~ 34.01 kg/m^2^), waist circumference, BP, total, LDL-, and HDL-cholesterol, FFA, glucose, insulin, HOMA-IR index, and Lp-PLA_2_ activity (Table 1). The change in body weight in the MCD group was -8.87% (-6.77±0.37 kg) (*P*<0.001; three-year follow-up compared with the baseline), whereas the change in the control group was 0.20% (0.14±0.15 kg). At the three-year follow-up, the control group had increased waist circumferences, whereas the MCD group had increased HDL-cholesterol and decreases in BMI, TG, insulin, and HOMA-IR index compared with that of baseline levels (Table 1). The changes in BMI, waist circumference, TG, insulin, and HOMA-IR index observed in the MCD group were significantly different from those in the control group after adjusting for baseline levels. The estimated total caloric intakes at the baseline were 2,347±59 kcal/d and 2,391±58 kcal/d in the control and MCD groups (*P*=0.593), respectively. At the three-year follow-up, the estimated total caloric intakes were 2,338±56 kcal/d and 2,194±57 kcal/d for the control and MCD groups (*P*=0.045), respectively. The MCD group had a greater reduction in estimated total caloric intake compared with that of the control group (-181±29 compared with -37±31 kcal/d; *P*=0.001). There were no statistically significant differences in the percentage of total caloric intake from macronutrients, especially the polyunsaturated/monounsaturated/saturated (P/M/S) fat intake ratio between the baseline (control, 1/1.11/0.77; MCD, and 1/1.12/0.75) and three-year follow-up (control, 1/1.10/0.76; MCD, and 1/1.11/0.73). There were no statistical differences in the TEE and percentage of participants who smoke and/or drink alcohol between the baseline and three-year follow-up (data not shown).

**Table 1:** Clinical characteristics of participants at baseline and at the end of the three-year clinical intervention period.

	Baseline		P^a^	Follow-up		P^b^	Change		P^c^	P^d^
	Control (n=47)	MCD (n=47)		Control (n=47)	MCD (n=47)		Control (n=47)	MCD (n=47)		
Weight (kg)	74.46 ±1.51	76.35±1.50	0.378	74.61±1.49	69.58±1.44^***^	0.017	0.14±0.15	-6.77±0.37	<0.001	<0.001
Body-mass index (kg/m^2^)	27.54±0.22	27.78±0.28	0.500	27.60±0.22	25.31±0.27^***^	<0.001	0.06±0.06	-2.48±0.14	<0.001	<0.001
Waist (cm)	85.69±0.75	87.47±1.03	0.167	89.19±0.67^***^	87.95±0.82	0.248	3.49±0.79	0.48±0.77	0.007	0.021
Systolic BP (mmHg)	121.06±1.97	123.43±1.90	0.390	122.32±1.85	125.74±2.05	0.218	1.26±1.73	2.32±2.06	0.694	0.361
Diastolic BP (mmHg)	74.74±1.46	76.04±1.60	0.550	75.45±1.54	76.51±1.49	0.621	0.70±1.45	0.47±1.40	0.908	0.850
Total-cholesterol (mg/dL)^#^	203.96±4.55	196.36±4.95	0.267	202.74±4.87	196.38±5.21	0.336	-1.21±5.07	0.02±3.40	0.840	0.803
LDL-cholesterol (mg/dL)^#^	129.57±4.77	125.82±4.92	0.664	122.88±4.44	126.99±4.53	0.621	-6.69±4.88	1.17±3.29	0.185	0.225
HDL-cholesterol (mg/dL)^#^	44.51±1.74	41.83±1.61	0.205	45.74±1.57	46.68±1.82^**^	0.786	1.23±1.24	4.85±1.42	0.058	0.116
Triglyceride (mg/dL)^#^	149.40±13.80	143.57±10.85	0.860	170.62±14.58^*^	113.57±8.70^**^	0.001	21.21±11.12	-30.00±9.29	0.001	<0.001
Free fatty acid (mEq/L)^#^	550.64±39.13	543.94±30.70	0.942	546.49±38.41	481.79±28.82	0.224	-4.15±37.23	-62.15±35.76	0.264	0.157
Glucose (mg/dL)^#^	93.89±1.28	96.55±1.63	0.238	94.47±1.46	96.40±1.65	0.408	0.63±1.24	-0.15±1.39	0.677	0.958
Insulin (mIU/mL)^#^	10.07±0.61	10.33±0.60	0.665	10.43±0.82	7.56±0.52^***^	0.011	0.36±0.64	-2.76±0.57	<0.001	<0.001
HOMA-IR^#^	2.32±0.15	2.51±0.17	0.403	2.44±0.20	1.83±0.14^***^	0.025	0.08±0.16	-0.67±0.16	0.001	0.002
Lp-PLA_2_ activity (nmol/mL/min)^#^	28.41±0.95	30.16±1.04	0.237	28.48±0.84	31.14±1.21	0.151	0.07±1.21	0.98±1.37	0.621	0.118

Mean ± SE.

# tested by logarithmic transformation. *P^a^*, values derived from independent *t*-test in baseline. *P^b^*, values derived from independent *t*-test in follow-up. *P^c^*, values derived from independent *t*- test in changed value. *P^d^*, values derived from independent *t*-test in changed value after adjustment for baseline.

* *P*<0.05,

** *P*<0.01,

*** *P*<0.001 derived from paired *t*-test. HOMA-IR = [Fasting insulin (μIU/mL) × Fasting glucose (mmol/L)] / 22.5.

### Non-targeted metabolic pattern analysis

**Figure 1.**
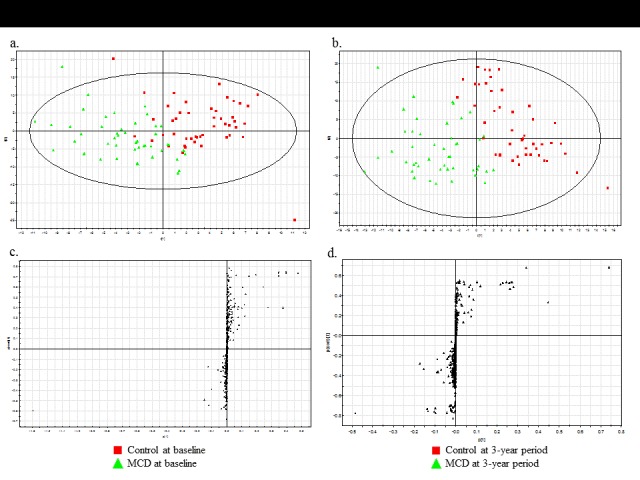
**Non-targeted metabolic pattern analysis**. (**A**) Score plots from PLS-DA models for the control at the baseline (*n*=47) and the MCD at the baseline (*n*=47). (**B**) The score plots from PLS-DA models for the control at the three-year follow-up (*n*=47) and the MCD at the three-year follow-up (*n*=47). (**C, D**) *S*-plots for covariance [*p*] and reliability correlations [*p*(corr)] from PLS-DA models.

**Figure 2.**
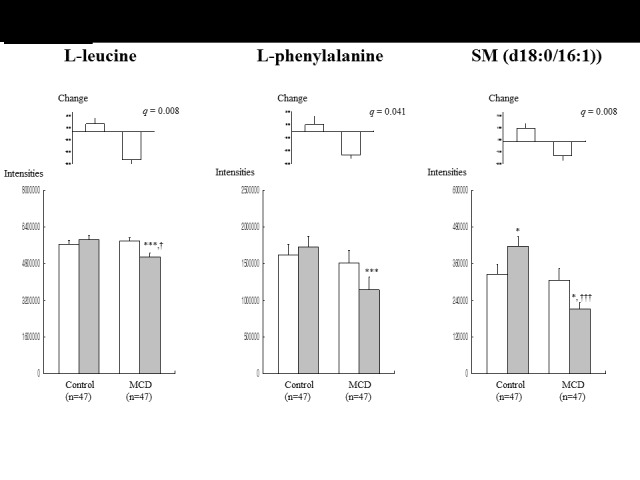
**L-leucine, L-phenylalanine, and SM (d18:0/16:1) at the baseline (□) and three-year follow-up (▢) in control and MCD individuals**. Normalized peak intensities ± SE; the changes are different from baseline values. ^*^q<0.05, ^**^q<0.01, ^***^q<0.001 compared with baseline values in each group. *^†^q*<0.05, *^††^q*<0.01, *^†††^q*<0.001 compared between two groups at the three-year follow-up.

The MS data for plasma metabolites obtained at the baseline and three-year follow-up were analyzed with PLS-DA score plots. A PLS-DA was conducted for the following combinations of groups: (1) the control group and the MCD group at baseline (Fig 1A), and (2) the control group and the MCD group at the three-year follow-up (Fig. 1B). There was no difference between the two groups at the baseline in their metabolic profiles, and the PLS-DA score plots showed neither a clear separation nor distinct clustering [*R*^2^*X*(cum)=0.157, *R*^2^*Y*(cum)=0.62, *Q*^2^*Y*(cum)=0.0715] (Fig. 1A). However, the two-component PLS-DA score plots of control and MCD at three-year follow-up showed distinct clustering and clear separation [with *R*^2^*X*(cum)=0.198, *R*^2^*Y*(cum)=0.731, *Q*^2^*Y*(cum)=0.554] (Fig. 1B). The separation between data for the three-year follow-up with and without MCD strongly indicated that the metabolomic pattern is altered by dietary intervention. The PLS-DA model was validated by a permutation test in both combinations of groups [(1) *R^2^Y* intercept = 0.454 and *Q^2^Y* intercept = -0.0766; (2) *R*^2^*Y* intercept = 0.463 and *Q*^2^*Y* intercept = -0.107]. To identify the metabolites that differentially determined the data at the baseline and three-year follow-up, *S*-plots of *p*(1) and *p*(corr)(1) were generated by using centroid scaling (Fig. 1C and D). The *S*-plots revealed that the metabolites with higher or lower *p*(corr) values served as the more relevant ions for discriminating between the two groups.

**Table 2:** Identification of plasma metabolites at baseline and at the end of the three-year clinical intervention period.

Identity	Formula	Exact Mass (M+H) ^2^amu	Observed Mass (M+H) ^2^amu	Normalized peak intensities				^1^VIP		
				Control (n=47)		MCD (n=47)		Baseline versus follow-up		3 years
				Baseline	Follow-up	Baseline	Follow-up	Control	MCD	Control versus MCD
Palmitic amide	C_16_H_33_NO	256.2640	256.2620	452412 ±64601	479348 ±58151	552611 ±88831	1025072 ±99972^**^^,^^†††^	0.3872	2.4670	2.5264
Oleamide	C_18_H_35_NO	282.2797	282.2778	3493063 ±427115	3693334 ±356799	4383329 ±470722	6112663 ±394130^*^^,^^†††^	2.6189	8.5897	11.1056
LysoPC (16:1)	C_24_H_48_NO_7_P	494.3247	494.3207	1159145 ±76414	818604 ±51901^**^	1146221 ±73203	881175 ±57299^*^	1.6500	1.2272	0.3318
LysoPC (16:0)	C_24_H_50_NO_7_P	496.3403	496.3365	14041375 ±572185	10890944 ±335717^***^	14208427 ±587524	12334692 ±454096^**^	15.2491	8.6487	7.3860
LysoPC (17:0)	C_25_H_52_NO_7_P	510.3560	510.3524	782824 ±52629	499767 ±28730^***^	749587 ±56540	502771 ±27867^***^	1.3792	1.1782	0.1044
LysoPC (18:2)	C_26_H_50_NO_7_P	520.3403	520.3364	4814444 ±213686	4183528 ±175446^*^	4902597 ±209988	4346160 ±135047^*^	3.3271	2.5860	1.1684
LysoPC (18:1)	C_26_H_52_NO_7_P	522.3560	522.3513	5037464 ±237241	3992332 ±167208^**^	4946123 ±222731	4160407 ±153681^*^	5.1127	3.6578	1.1740
LysoPC (18:0)	C_26_H_54_NO_7_P	524.3716	524.3678	8458738 ±402181	6851545 ±268175^**^	8050071 ±435066	6189394 ±228378^***^	7.8056	8.6314	3.4312
LysoPC (20:4)	C_28_H_50_NO_7_P	544.3403	544.3363	1495015 ±70462	1228705 ±51557^**^	1486131 ±66078	1254837 ±53157^**^	1.3050	1.0721	0.2438
LysoPC (20:3)	C_28_H_52_NO_7_P	546.3560	546.3521	727631 ±40937	512704 ±26288^***^	661323 ±34714	508098 ±30939^**^	1.0437	0.7094	0.2232
LysoPC (22:6)	C_30_H_50_NO_7_P	568.3403	568.3362	1021202 ±66649	754469 ±41746^**^	1066554 ±61876	838209 ±40089^**^	1.2994	1.0725	0.3958
PC (16:0/18:2)	C_42_H_80_NO_8_P	758.5700	758.5645	1358996 ±250724	1981688 ±387017	1131627 ±203078	1778197 ±357441	3.0264	3.5135	4.3346
PC (16:0/20:5)	C_44_H_78_NO_8_P	780.5543	780.5493	873905 ±69441	908976 ±68495	907979 ±119802	986724 ±147682	0.6923	0.7440	1.9820
PC (18:2/18:2)	C_44_H_80_NO_8_P	782.5700	782.5651	3340338 ±199641	4013326 ±214154^*^	3310428 ±15511	4130013 ±245147^*^	3.8458	4.2191	0.5501
Lactosylceramide (d18:1/12:0)	C_42_H_79_NO_13_	806.5630	806.5641	3834674 ±202341	4438920 ±202688	3817039 ±345179	4571838 ±243753	3.4856	3.5729	0.7901
PC (18:0/20:4)	C_46_H_84_NO_8_P	810.6013	810.5955	720491 ±49861	586684 ±31414^*^	846008 ±62564	641669 ±44677^*^	0.6498	1.1362	0.2496

Mean ± SE.

*q<0.05,

**q<0.01,

***q<0.001 derived from paired *t*-test.

†*q*<0.05,

††*q*<0.01,

†††*q*<0.001 derived from independent *t*-test in follow-up. ^1^VIP, Variable Important in the Projection. ^2^amu, atomic mass units.

### Identification of plasma metabolites

Among the 699 plasma metabolites, the metabolites that played influential roles in the separation between the groups were selected according to the parameter ‘Variable Important in the Projection’ (VIP), with VIP values over 1.0 indicating a high relevance for the difference between the two groups. 73 metabolites were selected based on VIP values over 1.0; of these, 19 were identified (54 were unidentified). The results of 19 identified plasma metabolites are shown in Table 2 (16 metabolites) and Fig. 2 (3 metabolites). There were no significant differences in the baseline metabolites between control and MCD groups. The three-year follow-up of the control group identified the following metabolite changes: 10 metabolites significantly decreased, including lysophosphatidylcholines (lysoPCs) (C16:1, C16:0, C17:0, C18:2, C18:1, C18:0, C20:4, C20:3, and C22:6) and phosphatidylcholine (PC) (18:0/20:4) (Table 2); and 2 metabolites significantly increased, including sphingomyelin (SM) (d18:0/16:1) (Fig. 2) and PC (18:2/18:2) (Table 2). The three-year follow-up of the MCD group identified the following metabolite changes: 13 metabolites significantly decreased, including L-leucine, L-phenylalanine (Fig. 2), lysoPCs (C16:1, C16:0, C17:0, C18:2, C18:1, C18:0, C20:4, C20:3, and C22:6), PC (18:0/20:4) (Table 2), and SM (d18:0/16:1) (Fig. 2); and 3 metabolites significantly increased, including palmitic amide, oleamide, and PC (18:2/18:2) (Table 2). Then we compared metabolite changes (differences from the baseline) between the control and MCD groups. The MCD group showed greater decreases in L-leucine (*q*=0.008), L-phenylalanine (*q*=0.041), and SM (d18:0/16:1) (*q*=0.008) compared with the control group (Fig. 2). At the three-year follow-up, the MCD group showed lower peak intensities of L-leucine and SM (d18:0/16:1) (Fig. 2) and higher peak intensities of palmitic amide and oleamide (Table 2), compared with the control group. There was no remarkable gender effect on the metabolites in both male and female participants.

### Correlation between changes in the BMI, biochemical parameters, and major plasma metabolites

**Figure 3.**
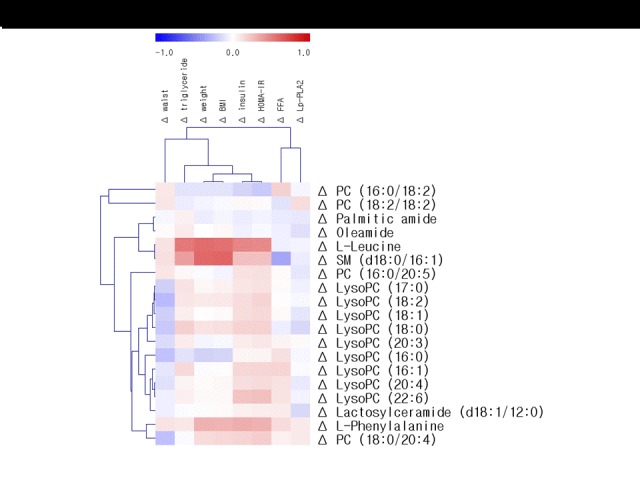
**Correlation matrix of changes (Δ) in metabolites and conventional risk factors in all subjects**. The supervised hierarchical clustering plot shows that the 20 most important metabolites stratify the samples according to conventional risk factors. Correlations were obtained by deriving a Spearman correlation coefficient. Metabolites are listed on the *left side* of the heat map, with conventional risk factors listed across the *top*. *Red* is a positive correlation and *blue* is a negative correlation.

In all subjects (*n*=94), the change (Δ) in BMI was positively correlated with a Δwaist circumference (*r*=0.265, *P*=0.010), ΔTG (*r*=0.501, *P*<0.001), Δinsulin (*r*=0.396, *P*<0.001), ΔHOMA-IR (*r*=0.396, *P*<0.001), ΔL-leucine (*r*=0.559, *P*<0.001), and ΔSM (d18:0/16:1) (*r*=0.602, *P*<0.001). Based on these results, we performed a multiple regression analysis to determine the independent predictors of ΔBMI. The age, gender, baseline BMI, Δwaist circumference, ΔTG, Δinsulin, ΔHOMA-IR, ΔL-leucine, and ΔSM (d18:0/16:1) were tested. Changes in the L-leucine (standardized *β*=0.262, *P*=0.005) and ΔSM (d18:0/16:1) (standardized *β*=0.375, *P*<0.001) emerged as independent predictors of ΔBMI, as did the Δwaist circumference (standardized *β*=0.208, *P*=0.008) and ΔTG (standardized *β*=0.175, *P*=0.049). Additionally, the ΔSM (d18:0/16:1) was positively correlated with ΔTG (*r*=0.383, *P*<0.001) and Δinsulin (*r*=0.250, *P*=0.015), and negatively correlated with ΔFFA (*r*=-0.349, *P*<0.001) and ΔlysoPC (16:0) (*r*=-0.308, *P*=0.003). The ΔBMI and Δweight were strongly and positively correlated with the ΔL-leucine, ΔSM (d18:0/16:1), and ΔL-phenylalanine in all subjects (Fig. 3 & Suppl. Fig. 1).

Supplementary Figure 1. **Correlation scatter plots of changes (Δ) in metabolites and conventional risk factors in all subjects**. *Circle* is a control group and *asterisk* is a MCD group.

### Correlation between changes in the HOMA-IR, biochemical parameters, and major plasma metabolites

In all subjects (*n*=94), the ΔHOMA-IR was positively correlated with the ΔBMI (*r*=0.396, *P*<0.001), ΔTG (*r*=0.400, *P*<0.001), Δfasting glucose (*r*=0.377, *P*<0.001), ΔL-leucine (*r*=0.460, *P*<0.001), ΔL-phenylalanine (*r*=0.305, *P*=0.003), and ΔSM (d18:0/16:1) (*r*=0.247, *P*=0.017). Based on these results, we performed a multiple regression analysis to determine the independent predictors of the ΔHOMA-IR. The age, gender, ΔBMI, baseline HOMA-IR, ΔTG, Δinsulin, Δglucose, ΔL-leucine, ΔL-phenylalanine, and ΔSM (d18:0/16:1) were tested. Changes in L-leucine (standardized *β*=0.241, *P*=0.033) emerged as an independent predictor of ΔHOMA-IR, as did baseline HOMA-IR (standardized *β*=-0.292, *P*=0.002) and Δglucose (standardized *β*=0.256, *P*=0.005). The ΔHOMA-IR, Δinsulin, and ΔTG were strongly and positively correlated with ΔL-leucine in all subjects (Fig. 3 & Suppl. 1).

### DISCUSSION

For the middle-aged overweight and obese participants of this study, a three-year intervention involving an MCD (an approximately 100 kcal/day deficit) resulted in a weight reduction of approximately 8.87%. Considering the age-related alterations of metabolites that were identified in this study, we identified 3 metabolites that showed statistically significant differences between the control and MCD groups, including L-leucine, L-phenylalanine, and SM (d18:0/16:1). The MCD group had greater decreases in L-leucine (*q*=0.008), L-phenylalanine (*q*=0.041), and SM (d18:0/16:1) (*q*=0.008), and the control group showed increases only in SM. At the three-year follow-up, 26 participants were no more overweight according to their BMIs. However, there was no significant difference in the final metabolite profile between lean and still overweight participants at the three-year follow-up in the MCD group (L-leucine, *q*=0.804; L-phenylalanine, *q*=0.434; and SM (d18:0/16:1), *q*=0.132; which decreased in both participants). This result is different from those of other studies suggested that the amino acids in obese increase in comparison with lean subjects. However, they were obese, not overweight, with a BMI of 36-37 kg/m^2^ on average [5, 6]. However, in this study, even some participants remained overweight at the three-year follow-up in the MCD group, and they were not extremely obesity as in previous studies. For that reason, there was no significant difference in the final metabolite profile between lean and overweight participants at the three-year follow-up in the MCD group.

The decreased level of SM (d18:0/16:1) in the MCD group is similar to that of a recent weight-loss study of an 8-week LCD in overweight and moderately obese women [22]. In terms of plasma lipoproteins, SM is the second-most abundant polar lipid after PC [22]. The proportion of plasma SM increases with age, and it is elevated in an obese model [23-26]. Thus, the increased SM level observed in the control group of this study could be related to aging. These results could suggest a preventive role for long-term mild caloric restriction on age-related increases in specific metabolites, particularly SM.

The current data indicate a positive correlation between changes in the HOMA-IR and changes in plasma L-leucine and L-phenylalanine. These results confirm a previous report showing that decreases in branched-chain and aromatic amino acids are significantly associated with decreased HOMA-IR and weight loss in overweight or obese subjects [27]. Studies of BCAA supplementation in both humans [28] and animals [29] demonstrate that circulating amino acids may directly promote IR through obstruction of insulin signaling in skeletal muscle. Append to IR, impaired insulin secretion has a critical role in the development of diabetes, in hoc, it is noticeable that BCAAs are modulators of insulin secretion [6, 29, 30]. Thus, another possible mechanism by which hyperaminoacidemia could promote diabetes is via hyperinsulinemia leading to pancreatic beta cell exhaustion. The research by Wang et al. [13] supported the concept that hyperaminoacidemia, particularly the BCAAs and phenylalanine, could be a very early manifestation of IR. Additionally, weight loss results in a fall in insulin level and a concomitant reduction in the BCAAs and phenylalanine [31]. Changes in the HOMA-IR also positively correlate with changes in the SM (d18:0/16:1). Hanamatsu et al. [32] demonstrate that the high levels of serum SM species with distinct saturated acyl chains including C18:0 closely correlate with the HOMA-IR and BMI. These results suggest that BCAAs, aromatic amino acids, and SM, as observed in our study as L-leucine, L-phenylalanine, and SM (d18:0/16:1), are associated with the development of IR. Therefore, decreased levels of these 3 metabolites after dietary intervention in our study may explain the preventive effects of long-term mild caloric restriction on IR. Cazzola et al. [22] investigated the effects of LCD on erythrocyte membrane properties, and they suggested that the decreased SM in the erythrocyte membrane composition of the LCD-induced weight loss group could reflect a virtuous cycle resulting from the reduction in IR associated with increased membrane fluidity that, in turn, results in a sequence of metabolic events that concur to further improve membrane fluidity.

With contributions of amino acids and SM (d18:0/16:1) to development of IR, it is well known that IR significantly affects lipoprotein metabolism and is associated with an increase in TG levels, decreased HDL levels, and an increase in the number of small dense LDL particles [33, 34]. Eckel et al. [35] suggested that a major contributor to the development of IR is an excessive circulating fatty acids. However, the situation is not only FFA elevation but TG concentrations are also increased [36]. The result of present study was consistent with previous research which shows strongly positive correlation between changes in IR and TG. In addition, after three-year follow-up, TG, HDL-cholesterol, insulin, and HOMA-IR were decreased in MCD group, whereas FFA did not show any changes after dietary intervention. Therefore, the current data indicate that the improvement of HOMA-IR during intervention is resulting from a decreased TG and increased HDL-cholesterol rather than changes in FFA.

This study indicates that changes in the SM (d18:0/16:1) are an independent, positive predictor of BMI changes, as are changes in waist circumference, TG, and L-leucine. A positive correlation between changes in the TG and SM (d18:0/16:1) is consistent with the previous report [37]. The negative correlation between changes in the FFA and SM could suggest an inhibitory effect of the SM on the lipoprotein lipase activity [23]. Therefore, this result may indicate the beneficial effects of a long-term diet-induced weight reduction on phospholipid metabolism, because a higher SM level is a marker of abnormal sphingolipid metabolism and a possible risk factor in atherosclerosis [26, 37]. However, Mamtani et al. [38] found the inverse correlation of two SM species (31:1 and 41:1) with waist circumference. Schwab et al. [39] also detected a modestly but not significantly elevated SM in the weight-reduction group after a 33-week dietary intervention in overweight-obese individuals. These contradictory results may be explained in part by differences in experimental approaches and/or study populations (16 participants compared with 94 participants), age range (40-70 years compared with 40-59 years), intervention period (33 weeks compared with 3 years), and weight-loss amount (-7.8% compared with -8.87%).

Hojjati et al. [40] found that a decreased plasma SM level and an increased PC level have major roles in the prevention of atherosclerosis. Schwab et al. [39] reported a decrease in the PC (18:0/20:4) and no change in the lysoPC (16:0) after 33 weeks of dietary intervention. However, the current study identified an age-related increase in the PC (18:2/18:2), combined with decreases in the PC (18:0/20:4) and all 9 lysoPCs (C16:1, C16:0, C17:0, C18:2, C18:1, C18:0, C20:4, C20:3, and C22:6) in both the control and MCD groups. This result is consistent with the results of a previous animal study, in which decreases in lysoPCs were observed with advancing age [41). LysoPCs, which represent 5-20% of the total plasma phospholipids, are usually generated from the PC present in lipoproteins by PLA_2_ or by LCAT [42]. Lp-PLA_2_, which is primarily bound to LDL-cholesterol, catalyzes the hydrolysis of the ester bond at the *sn*-2 position and produces bioactive oxidized FFAs and lysoPCs [43, 44]. No age-related changes in the Lp-PLA_2_ activity and LDL-cholesterol were observed; therefore, the age-related decreases in all the lysoPCs of both groups were not related to Lp-PLA_2_ but to a decrease in PLA_2_-induced hydrolysis or LCAT activity. Recently, age-related decreases in lysoPC (16:1 and 18:4) levels were restored by a 40% caloric restriction in aged mice [41]. In current study, the MCD group showed less reduction in lysoPC (16:1, 16:0, 22:6) levels compared with those of the controls, but these differences were not statistically significant.

Current study has several limitations. First, we specifically focused on a representative group of South Koreans aged 40-59 years. Therefore, our data must be further explored to be generalized to other ethnic groups or counties/populations. Second, a dietary intake was based on self-reports obtained from weighed food. However, measurement errors from self-reported dietary intake and lifestyle variables have been shown to be relatively small [45, 46]. Third, although a large number of metabolites were detected by UPLC-LTQ-Orbitrap MS in this study, most of them are currently unidentified. Large databases of endogenous biomolecules have not been constructed yet for use with LC-MS-based techniques for metabolomics research [47]. Fourth, the relatively small sample size used in this study may not be sufficiently large to detect all long-term MCD-associated metabolic changes. Despite these limitations, our approach of using UPLC-LTQ-Orbitrap MS-based metabolomics and multivariate data analysis revealed a greater reduction in L-leucine, L-phenylalanine, and SM (d18:0/16:1) in the MCD group during three-year mild caloric restriction compared with that of the control group. Our data indicate a protective role of long-term mild caloric restriction against age-related increases in specific metabolites, particularly SM. The reduction in L-leucine and L-phenylalanine can provide valuable clues about the mechanism underlying decreased IR following diet-induced weight reduction, even classical lipoprotein measures are not significantly altered.

We examined the effect of weight loss from a long-term MCD on plasma metabolites, and we identified 3 metabolites that showed significant differences between the control and MCD groups. The MCD group showed greater decreases in L-leucine, L-phenylalanine, and SM than those of the control group. In considering that the proportion of plasma SM increases with age, these results could suggest a preventive role for long-term mild caloric restriction in overweight and obese subjects for age-related increases in specific metabolites, particularly SM. There were positive correlations between the changes in HOMA-IR and the changes in plasma L-leucine, L-phenylalanine, and SM. These results suggest that the reduction of L-leucine and L-phenylalanine, which are indicated as decreased levels of 3 metabolites, may explain the remedial effects of long-term mild caloric restriction on IR. A positive correlation between the changes in TG and SM may indicate the beneficial effects of MCD-induced weight reduction on phospholipid metabolism because a higher SM level is a marker of abnormal sphingolipid metabolism and a possible risk factor in atherosclerosis.
